# The Development of Leisure Participation Assessment Tool for the Elderly

**DOI:** 10.1155/2020/9395629

**Published:** 2020-12-04

**Authors:** Eun-Hwa Jeong, Eun-Young Yoo, Jong-Bae Kim, Jung-Ran Kim, Dae-Sung Han, Ji-Hyuk Park

**Affiliations:** ^1^Dept. of Occupational Therapy, College of Health Science, Far East University, 27601, 76-32, Daehak-gil, Gamgok-myeon, Eumseong-gun, Chungcheongbuk-do, Republic of Korea; ^2^Dept. of Occupational Therapy, College of Health Science, Yonsei University, 26493, 1, Yeonsedae-gil, Heungeop-myeon, Wonju-si, Gangwon-do, Republic of Korea; ^3^Dept. of Dementia Prevention and Rehabilitation, College of Human Service, Catholic Kwandong University, 25601, 24, Beomil-ro 579beon-gil, Gangneung-si, Gangwon-do, Republic of Korea; ^4^Dept. of Occupational Therapy, College of Health Science, Sangji University, 26339, 84, Sangjidae-gil, Wonju-si, Gangwon-do, Republic of, Republic of Korea

## Abstract

**Objectives:**

This study is aimed at developing multidimensional leisure participation assessment tool for the elderly to achieve quantitative and qualitative assessment of leisure participation and leisure exploration.

**Methods:**

This study collected preliminary items through literature review, statistical office data, and survey of the elderly's leisure activities and considered the list of leisure activities as assessment items by conducting a Delphi survey. Reliability was verified through internal consistency and test-retest reliability. The assessment tool was finally confirmed using content validity and discriminant validity.

**Results:**

A total of 81 leisure items classified into 8 categories and 22 subcategories were obtained through data collection and Delphi survey. Cronbach's *α* value was 0.939, and Intraclass Correlation Coefficient was 0.941. A content validity test was confirmed by validating that I-CVI was 0.78 or more and the S-CVI was 0.95. According to the result of discriminant validity, there was a difference in the number of participating leisure activities and leisure activities with participation intention by age.

**Conclusion:**

The leisure participation assessment tool for the elderly developed in this study can obtain information on the overall view of the leisure of the elderly by measuring leisure exploration, leisure participation, and interference factor affecting leisure participation.

## 1. Introduction

Leisure is defined as a “Nonobligatory activity that is intrinsically motivated and engaged in during discretionary time, that is, time not committed to obligatory occupations such as work, self-care, or sleep” [1, 2]. Leisure time varies according to human life cycle. Especially in old age, life after retirement shifts from work to leisure and has more time than previous life.

Statistics Korea has been conducting a survey on the lifetime of citizens in order to measure their lifestyle and time use from 1999 onward and provides basic data on the leisure time of Korean elderly. According to the Statistics Korea's Life Time Survey, young adults in their 20s and 30s spent approximately 100 min per weekday on leisure activities, those in their 60s spent 234.6 min per weekday, and those in their 70s spent 287.8 min per weekday [3]. However, the proportion of the elderly not prepared for leisure was 49.2% despite the increase in leisure time for the elderly [4, 5]. The reason for this is that elderly men have lived focus on their works before the retirement, so they lacked the experience of exploring and participating in various meaningful leisure activities. And elderly women are reported to feel difficulty in using leisure time because they have been living in a family-centered manner [4, 6]. Leisure is one of the occupational areas that is mostly occupied by the elderly during the day. Occupational therapists should consider leisure as a special and important area in the elderly's lives. In community-based occupational therapy, leisure activities are consistently being developed and an academic basis for the improvement of elderly's leisure participation has been established [7–10]. However, there is no standardized assessment tool that assesses the elderly's leisure participation in occupational therapy [5, 7]. According to Jeong et al.'s [5] systematic review of leisure participation assessment tools for the elderly, leisure assessment tools used in the 2000s were classified as Activity Card Sort (ACS) and Physical Activity Scale for the Elderly (PASE). Moreover, most of the studies have been using self-developed assessment tools since 2010.

ACS is a representative evaluation tool for assessing the elderly's level of activity which consisted of 80 items in four areas including instrumental activities, social activities, and low-intensity and high-intensity leisure activities [11]. In 2010, the Korean Activity Classification Card (K-ACS) was developed comprising activities reflecting Korean culture, and it is used as a leisure assessment tool for Korean elderly. Although K-ACS has the advantage that it is composed of items that reflect the Korean culture regarding leisure activities in the elderly, it mainly consisted passive activities such as “Watching TV” and “Listening to the radio.” There is a limit to the lack of diversity of leisure activities that consist of low-intensity activities [5, 12]. Furthermore, K-ACS has the disadvantage that leisure activities insufficiently vary and only comprise low-intensity activities [5, 12].

PASE is an assessment for measuring the level of physical activity of the elderly over 65 years old. It consists of 75 items of housework and leisure activities [13]. PASE includes housework, strength, and endurance items other than leisure, which limits its use as leisure-oriented assessments. In addition, the PASE assessment method can only quantitate aspects of each activity by measuring the performance of the activity during the day (rarely/occasionally/often). Therefore, it is difficult to identify qualitatively about whether leisure participation is meaningful and positive for the individual, such as how satisfied or unsatisfied they are with the current leisure participation [5, 13].

The measurement of participation should include both quantitative outcomes of objective data and personal outcomes of subjective experience. The multidimensional measurement of the participation evaluation tool has the advantage that it is possible to qualitatively judge not only the objective state but also meaningful leisure participation by including not only performance and frequency of participation but also satisfaction or interest [14]. Measuring qualitative factors such as satisfaction or interest allows the measurement of clients' perceptions of their participation, evaluating their level of involvement in the occupation and the value of their occupation [15, 16]. The measurement of qualitative factors reflects individual values, so it is an important measurement factor as it facilitates client-centered intervention. Therefore, in order to effectively mediate the leisure time of the elderly, the elderly's leisure participation assessment should be multidimensional measurement for the quantitative and qualitative assessment of leisure participation (Kim, Jung, Yoo, Park, Kim & [17, 18]).

In the Occupational Therapy Implementation System (OTPF), there are leisure exploration to identify leisure, interests, skills, opportunities, and appropriate leisure activities and leisure participation to plan and participate in appropriate leisure activity participation [1]. This includes not only participating in leisure but also exploring leisure activities that can lead to healthy and beneficial old age. Therefore, it is necessary to develop an evaluation tool for elderly leisure participation that can obtain overall information on leisure exploration and participation of the elderly from an occupational therapy perspective and measure all aspects of quantitative and qualitative of leisure.

Therefore, the purpose of this study is to develop a multidimensional measurement tool for elderly leisure participation and leisure exploration that can quantitatively and qualitatively identify leisure time in order to expand the community-based occupational therapy service for the elderly.

## 2. Methods

**Figure 1:**
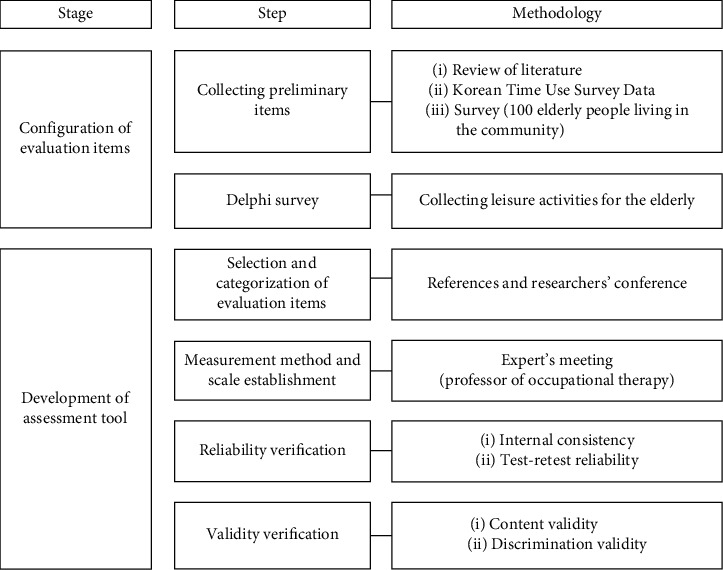
The development process of the leisure participation assessment tool for the elderly.

This study consisted of two stages. The first stage comprised the evaluation items of the leisure participation assessment tool for the elderly obtained through preliminary item collection and Delphi survey, and the second stage was the development of the leisure participation assessment tool for the elderly including the reliability and validity verification (Figure 1). This study was approved by Yonsei University Wonju Institutional Review Board of Wonju Campus of Yonsei University (1041849-021806-BM-055-01).

### 2.1. Configuration of Evaluation Items

#### 2.1.1. Collecting Preliminary Items

The literature review was conducted as the first step of data collection. The purpose of the literature review was to collect the evaluation items of the existing leisure assessment tools by examining the previous study conducting leisure assessment of the elderly. The databases used for the thesis search were SCOPUS, PubMed, NSDL, and RISS. Major search terms were retrieved by combining words such as “Old people,” “Elderly,” “Leisure,” “Assessments,” “Measurement,” and “Elderly leisure activities.” A total of 170 papers were searched. The research was reviewed by title or abstract and selected by checking the original text, if necessary. The final selected papers were 14, and the items of leisure evaluation tools used in 14 papers were extracted. And the items were collected excluding overlapping leisure activities.

The second step of collecting the preliminary items was to refer to the Korean Time Use Survey Data [3]. The Life Time Survey Behavior Classification [3] used in this study includes compulsory activities, including personal maintenance, work, learning, home management, care for family and household members, and mobility, and leisure activities including volunteer work, social participation and leisure activities. In this study, leisure activities based on OTPF were added among activities included in leisure activities. At this time, leisure activities that overlap with items collected in the literature review were excluded.

As the third step of collecting preliminary items, the elderly answered the open-ended questionnaire about leisure activities that they actually participated during their leisure time. The open-ended questions were as follows: (1) What leisure activities do you actually participate in during leisure time? (2) What leisure activities do you think most elderly people participate in when you think? The elderly people aged 65 years or elderly living in the community were never diagnosed with any disease physically, cognitively, or mentally and had no serious cognitive deficits. The collected responses were selected through the researchers' meetings except for the duplicate responses to the items collected through the literature review and the National Statistical Office data.

#### 2.1.2. Delphi Survey

Delphi survey was conducted as a research method for constructing evaluation items. The purpose of the Delphi survey was to collect comprehensive data on the elderly's leisure activities by conducting a Delphi survey of occupational therapists who can efficiently provide leisure activities considering the physical, cognitive, and emotional characteristics and needs of the elderly. Subjects of the Delphi survey included occupational therapists who have experience in community occupational therapy and who have expert opinion and knowledge regarding occupational therapy for the elderly. A total of 20 subjects understood the purpose of the study and agreed to participate in the study. The Delphi survey of this study was conducted in three phases through email. The first Delphi survey conducted an unstructured open-ended questionnaire to obtain expert panel opinions on leisure activities for the elderly. The questionnaire was about the leisure activities of Korean elderly. It included activities that can be performed in the house and around the house/neighborhood (within 10 km) and during institutional visits (welfare center, cultural center). The response to the elderly leisure activities collected by the first open-ended questionnaire was integrated with the preliminary item by the researcher, and the duplicate items were deleted. These items were selected for the second Delphi survey.

The second Delphi survey adequately assessed the appropriateness of the elderly's leisure activities collected in the first survey using the 5-point Likert scale (5, highly appropriate; 1, very inappropriate) and responded to further comments on the questions if necessary. For the data analysis, the content validity ratio (CVR), mean, standard deviation, stability, convergence, and consensus were analyzed using the 5-point Likert scale. The formula of content validity ratio was CVR = (Ne − *N*/2)/(*N*/2), in which Ne was the number of panelists indicating “essential” and *N* was the total number of panelists. The stability verification was measured by the coefficient of variation, which is the standard deviation divided by the mean. For the convergence degree, the formula of *Q*3 − *Q*1/2 was used, and for the consensus degree, the formula of 1 − (*Q*3 − *Q*1/Mdn) was used. When stability was 0.8 or less, convergence was 0.5 or less, and consensus was 0.75 or more, it was considered statistically significant [19].

The third Delphi survey was assessed using a 5-point Likert scale (5, highly appropriate; 1, very inappropriate) to assess the appropriateness of the final analysis items in the second Delphi survey. At this time, the mean, standard deviation, and median of the second Delphi survey were presented. So subjects responded by taking into account the opinions of the other subjects of the Delphi survey. The reasons for the responses were recorded for each item below 3 points. For the data analysis, the CVR, mean, standard deviation, stability, convergence, and consensus were analyzed using the 5-point Likert scale.

### 2.2. Development of Assessment Tool

#### 2.2.1. Selection and Categorization of Evaluation Items

The categorization of leisure activities for the elderly is based on a factor analysis study of leisure activities for the elderly living in the community by Iwasa and Yoshida [20]. It was intended to present various leisure activities of elderly by selection and categorization leisure activities of the elderly in Korea through a Delphi survey based on existing factorial analysis studies. It was based on 11 categories: physical, social-public, raising plants, intellectual game, social-private, competitive game, technology use, travel, creative, developmental, and cultural. Finally, the categorization of leisure activities was decided through the researchers' meeting.

#### 2.2.2. Measurement Method and Scale Establishment

The measurement method was based on the Canadian Model of Occupational Performance and Engagement (CMOP-E) and the Community Participation Indicator (CPI), which is a model that includes the concept of participation among occupational therapy execution models [21–23].

CMOP-E is an implementation model based on the concept of participation in occupational therapy. CMOP-E considers working participation to be facilitated by dynamic interactions among people, occupations, and environments [21, 23]. This study used the CMOP-E model as a tool to assess leisure participation in the elderly and to construct a measurement method that can identify the leisure participation and leisure exploration and related environmental factors of the elderly. A typical assessment based on the CMOP-E model is a measurement method that helps to develop client-centered interventions by measuring satisfaction, importance, or interest, such as Canadian Occupation Performance Measurement [24]. Moreover, the CPI is an assessment tool that measures the frequency and degree of participation in activities and the objective and subjective characteristics of participation [22]. Therefore, we determined multidimensional measurement and scales to enable quantitatively and qualitatively measure of leisure time in order to develop an assessment for participation and exploration for the leisure of the elderly from the viewpoint of occupational therapy.

#### 2.2.3. Reliability Verification

*(1) Internal Consistency*. To verify the internal consistency, 100 elderly people living in the community were enrolled in the completed leisure participation assessment tool for the elderly. The inclusion criteria were as follows: (1) the elderly people aged 65 years or elderly living in the community, (2) subjects who were never diagnosed of any disease physically, cognitively, or mentally and had no serious cognitive deficits, and (3) subjects who agreed to participate in the study. The researcher visited the elderly welfare center, senior center, or the local church to recruit the subjects. The evaluation consisted of a semistructured interview conducted by the researcher.

The data were analyzed using the Statistical Package for the Social Sciences (SPSS) version 20.0 and using 0.05 as the critical significant level. The internal consistency was analyzed using Cronbach's *α* coefficient for satisfaction or interest values. Cronbach's *α* coefficient has a value between 0 and 1. In general, the reliability criterion was acceptable when it was 0.6 or more and was even more reliable when it was 0.7 or more [25]. In the analysis, Cronbach's *α* values of each item, leisure participation, leisure exploration, and interference factors, were analyzed.

*(2) Test-Retest Reliability*. In the test-retest reliability test, 20 out of 100 subjects who completed the leisure participation assessment for the elderly of internal consistency test were repeatedly measured at 2-week intervals.

The data were analyzed using the Statistical Package for the Social Sciences (SPSS) version 20.0, and using 0.05 as the critical significant level. The test-retest reliability was analyzed using the intraclass correlation coefficient (ICC) for satisfaction or interest values. The test-retest analyzed it by setting a two-way mixed model and consistency type. ICC has a value from 0 to 1, and closer to 1 means higher reliability. Generally, 0.75 or more were considered to be highly reliable [26].

#### 2.2.4. Validity Verification

*(1) Content Validity*. Using the leisure participation assessment tool for the elderly developed in this study, the content validity was verified to confirm the categorization system of the evaluation item. We used the item-level content validity index (I-CVI) and scale-level content validity index (S-CVI) based on the studies of Lynn [27] and Polit and Beck [28]. I-CVI was a method that assesses how well the expert group reflects the concept of the item on a scale of 3 to 5 [27]. In this study, we constructed a 4-point scale (1 point, very inappropriate; 2 points, inappropriate; 3 points, appropriate; and 4 points, very appropriate). If you responded with 1 point (very inappropriate) or 2 points (inappropriate), you will be asked to comment on the reason. Based on a previous study [27], 10 community-based occupational therapists, adult occupational therapists, or occupational therapist professors with at least 3 years of occupational therapy experience were enrolled in this study. For the analysis, I-CVI was calculated as 1 point for questions with experts' results of 3 or 4 points and 0 point for questions with 1 or 2 points [29]. So, I-CVI was calculated by dividing the number of persons who answered 3 points (appropriate) or 4 points (very appropriate). S-CVI is for evaluating the validity of scale-level, and we analyzed by S-CVI/Averaging method in this study. S-CVI/Ave is the average of I-CVI [29]. So, S-CVI for all items was calculated as the I-CVI average of each item. A previous study suggested that I-CVI should be greater than 0.78 and S-CVI should be greater than 0.9 when there were 10 panel experts [28].

*(2) Discrimination Validity*. To verify the validity of leisure participation assessment tool for the elderly developed in this study, the validity of discrimination at the level of leisure participation in the elderly was verified by analyzing the results of 100 elderly residents living in the community by age. According to the previous studies, there is a tendency that the desire for a new leisure decreased due to increasing age and the lack of acceptance for new activities due to the rejection of new ones [30–32]. Hence, the analysis of the number of leisure activities and the number of leisure activities in which elderly people participated by age were analyzed using 22 subcategories of leisure participation assessment developed in this study. The contents of the analysis were analyzed by the number of participating leisure activities in three groups of the 60s, 70s, and 80s and the number of activities of leisure exploration of three groups. The data were analyzed using the Statistical Package for the Social Sciences (SPSS) version 20.0 and using 0.05 as the critical significant level.

## 3. Result

### 3.1. Configuration of Evaluation Items

#### 3.1.1. Collecting Preliminary Items

As a result of the literature review, which is the first step of collection of preliminary items, 13 leisure assessment tools for elderly were selected in 14 studies. A total of 353 leisure activities were collected by combining the leisure items of each assessment tool. We collected 57 items from the collected leisure activities by consolidating the activities with similar concepts and eliminating duplicate activities.

As a result of the Korean Time Use Survey Data, which is the second step of collecting the preliminary items, Most items were duplicated with the first step item. One item of “pleasure” was added, and 58 items were collected until the second step.

As a result of the survey on leisure activities for the elderly, which is the third step of collection of preliminary items, most items overlapped with items up to the second step. Three items were added: “Housework,” “Taking care of grandchildren,” and “Religious activities”. These activities were included in the activity of IADL or social participation in the occupational therapy practice framework, but these activities were included as items because they responded with their own leisure activities. Moreover, a previous study reported that the leisure of the elderly is not complicated and is composed of monotonous activities related to their life [31]. Therefore, a total of 61 items were extracted from the preliminary item collection, and this item was used as a basic data for constructing the list of leisure activities for the elderly using the Delphi survey.

#### 3.1.2. Delphi Survey

A total of 196 items were obtained from the first Delphi survey. To construct the items of the 2nd Delphi survey, 61 items derived from the collection of preliminary items and 196 items derived through the 1st Delphi survey were integrated, duplicate items were deleted. As a result, we obtained 114 leisure activities.

The second Delphi survey was designed to assess the appropriateness of leisure activity for the elderly using a 5-point Likert scale for 114 items collected in the first survey. As a result of analyzing the response value, we deleted 5 items (“Housework,” “Hunting,” “Drinking,” “Pleasure,” and “Walking on the beach”) analyzed by a CVR value less than 0.42. As a result, we obtained 109 items.

The third Delphi survey was designed to assess the elderly's leisure activities using a 5-point Likert scale for 109 items obtained from the second survey. As a result of analyzing the response value, we deleted 22 items analyzed by a CVR value less than 0.42. Deleted items were “Letter writing,” “Taking care of a grandchild,” “Attending a song festival,” “Poetry reading,” “Billiard,” “Barista class,” “Soccer,” “Baseball,” “Hill climbing,” “Bowling,” “Yuchnoli,” “Kendo,” “Pop art,” “Pilates,” “Nanta,” “Darts,” “Viewing the sunrise,” “Archery,” “Horse riding,” and “Hiking”.

The third Delphi survey showed higher average CVR, convergence, consensus, and stability than the second Delphi survey (Table 1). Thus, the final 87 leisure activities for the elderly were finally obtained from the results of the third Delphi survey (Table 2).

### 3.2. Development of Assessment Tool

#### 3.2.1. Selection and Categorization of Evaluation Items

Duplicate items were merged and deleted from the 87 items obtained from a preliminary item collection and Delphi survey through the researchers' meeting. Instrumental activities of daily livings considered cannot be noncompulsory to participate during free time such as “Spending time with pets,” “Taking a walk with pets,” “Cooking,” “Making healthy foods,” and “Joining resident association activities” were deleted. Therefore, a total of 81 items were finally determined as elderly's leisure activities.

Leisure activity categorization results were classified into 8 major categories of sports, games, social, cultural, learning, recreation, outing, and information communication based on Iwasa and Yoshida [20]. In addition, 8 major categories were classified into 22 subcategories according to the characteristics of their activities (Table 3).

#### 3.2.2. Measurement Method and Scale Establishment

The measurement of this assessment was determined as follows based on the CMOP-E and the CPI: (1) leisure participation—frequency of participation and satisfaction, (2) leisure exploration—frequency of participation and interest, and (3) interference factors—eight factors interfering with leisure participation (physical, economic, time, information, environmental, and attitude constraints).

The frequency of leisure participation was chosen based on the following: no, weekly, monthly, or annually. Then, the subject was required to enter the number of times they were participating. Similarly, the frequency of leisure exploration was chosen based on the following: no, weekly, monthly, or annually. The subject was then asked to enter the number of times they wanted to participate.

Satisfaction and interest were measured using a 10-point scale that allows subjects to directly score a client's present condition. The closer the score to 1, the more dissatisfied or less interested, and the closer the score to 10, the greater the satisfaction or the greater the interest.

The constraints that interfere with leisure participation based on the previous studies (Edginton, Hanson, & Edginton) were measured using a 5-point Likert scale (1, very likely; 2, somewhat likely; 3, neutral; 4, somewhat unlikely; and 5, very unlikely).

#### 3.2.3. Reliability Verification

*(1) Internal Consistency*. According to the result of the internal consistency analysis, the Cronbach's *α* value of the whole item was very high (0.939) (Table 4). Additionally, the value of Cronbach's *α* in leisure participation was 0.911, which showed very high reliability. The item-total correlations for each item ranged from -0.043 to 0.803 (Table 4). The value of Cronbach's *α* in leisure exploration was 0.921, which showed very high reliability. The item-total correlations for each item ranged from 0.626 to 0.942 (Table 4). The value of Cronbach's *α* in interference factors was 0.458, which showed low reliability (Table 4).

*(2) Test-Retest Reliability*. The results of the analysis showed that the ICC was high at 0.941 (0.897-0.972 [95% CI], *p* = 0.000) in all items and was statistically significant.

#### 3.2.4. Validity Verification

*(1) Content Validity*. To verify the validity of the categorization of the leisure participation assessment for the elderly item developed in this study, I-CVI was calculated. As a result, I-CVI of the 8 categories and 22 subcategories were 0.78 or more and were validated (Table 5). I-CVI for each item ranged from 0.80 to 1.00 (Table 5). S-CVI was 0.95 and was validated for the categorization of all items (Table 5). In the case of the content validity questionnaire, if one responded with 1 point (very inappropriate) or 2 points (inappropriate), the reason was considered to describe the expert opinion, and the results of the response were discussed through the researcher's meeting. Regarding the opinion that “fishing” of the “Game” category derived from the questionnaire was inappropriate, fishing is a sophisticated physical activity with sensorimotor characteristics, not just leisure activity, but professional knowledge of fish habitat, adjustment of fishing line length, etc. Since it was an activity that requires the skill or rule of, it was decided to be included in the relevant category [33]. Regarding the opinion that it is inappropriate to include the “horticultural activity” of the “Culture” category, it was decided to be included in the category of culture from the viewpoint of activities that have various effects that are also effective for stability. The “shopping” activity of the “Outing” category was included as a leisure activity for enjoyment, rather than a purposeful activity of daily activities. The “Information communication” category of “Watching television” and “Listening to the radio” was also included in each category as leisure activities for enjoyment rather than purposeful activities.

*(2) Discrimination Validity*. As a result of the analysis, the number of participating leisure activities in the 60s was 14.14 ± 3.58, the number of participating leisure activities in the 70s was 7.51 ± 3.31, and the number of participating leisure activities in the 80s and above was 5.09 ± 1.73 (Table 6). Additionally, the number of activities of leisure exploration in the 60s was 5.60 ± 5.16, the number of activities of leisure exploration was 1.90 ± 2.42 in the 70s, and the number of activities of leisure exploration was 1.12 ± 0.99 in 80s and above (Table 6). There was a significant difference in the number of activities of leisure participation and the number of activities of leisure exploration by age group of 60s, 70s, and 80s. As a result of the post hoc assessment of the number of leisure participation activities by age, the number of leisure participation activities was highest in the 60s, followed by 70s or 80s and above. As a result of the post hoc assessment of the number of activities of leisure exploration by age, the number of leisure exploration activity was highest in the 60s (Table 6).

## 4. Discussion

This study is aimed at developing a multidimensional leisure participation assessment tool for the elderly that can quantitatively and qualitatively measure the leisure time of the elderly in order to expand the community occupational therapy services for the elderly.

A total of 81 leisure items classified into 8 categories and 22 subcategories were obtained through data collection and Delphi survey. Internal consistency of the total items was high with 0.939 Cronbach's *α* value. Additionally, the test-retest reliability was found to be a highly reliable assessment with an intraday correlation coefficient of 0.941. A content validity test was confirmed by validating that the I-CVI of the categorization of the leisure participation assessment for the elderly item was 0.78 or more and the S-CVI was 0.95. According to the result of discriminant validity, there was a difference in the number of participating leisure activities and the number of activities of leisure exploration by age, and it was confirmed that leisure participation and leisure exploration decreased with age. Therefore, it can be deduced that this assessment has discriminative power in determining the level of leisure participation according to the age of the elderly.

In occupational therapy, assessment of participation in leisure activities is traditionally conducted in an unstructured method, and the standardized tool used does not reflect age or cultural characteristics [12]. This study is aimed at establishing an evaluation item of leisure activities for Korean elderly to explore leisure activities that can be considered in leisure participation mediation and at determining the participation status of the elderly. In this study, for preliminary items, we collected the leisure activities of domestic and foreign elderly through the review of literature, the National Statistical Office Life Time Survey classification, and the questionnaire. Additionally, Delphi survey was conducted to collect the opinions of occupational therapists, considered as experts who can select, analyze, and apply leisure activities appropriate for the elderly, resulting in the collection of preliminary items and organization of evaluation items. Thus, it could be observed that the elderly's leisure activities that reflect the characteristics of the domestic culture were comprehensively presented as evaluation items. Therefore, it was considered that the assessment developed in this study comprehensively presents the elderly's leisure activities reflecting the characteristics of the domestic culture and the elderly's age.

The categorization of leisure activities of the leisure participation assessment for the elderly developed in this study consisted of 8 categories and 22 subcategories of evaluation items based on the factor analysis study of the elderly's leisure activities living in the community in Iwasa and Yoshida [20]. A previous study has shown that the classification of productive leisure activities and consumption leisure activities is widely applied [34, 35]. Productive leisure activities include health, sports, hobbies, learning, religion, and social participation activities, and consumption leisure activities include media use, relaxation, entertainment, and social activities using any material or product as passive activities [34, 35]. The assessment for elderly's leisure participation developed in this study included activities that correspond to the classification of productive/consumption leisure activities and the type of leisure activities of Korean elderly of social, natural-oriented, static, and exercise [36]. Therefore, leisure participation assessment for the elderly developed in this study established the classification of leisure activities considered to be academically beneficial, which reflected the elderly's leisure activities. Content validity was also verified to confirm the validity of the categorization of leisure activities.

The tool developed in this study to evaluate the elderly's leisure participation consisted of multidimensional measurement, which included quantitative measures such as frequency and qualitative measures such as satisfaction and interest by supplementing the limitations of existing leisure assessments. Existing leisure assessments mainly measure the participation and frequency, and there are limitations in identifying meaningful leisure participation, such as how satisfied the elderly are in their leisure time or whether the elderly are interested in any leisure activity. Thus, it requires that the evaluation of the overall view of the elderly leisure that the positive leisure participation is achieved through the measurement of the qualitative aspect such as satisfaction or interest as well as the quantitative aspect, such as the frequency. Therefore, the tool developed in this study to evaluate the elderly's leisure participation consisted of measuring personal factors such as frequency of activity, satisfaction, and interest and environmental factors such as interference. Measurements of interference factors affecting leisure participation were included in terms of the environment affecting individual and leisure participation. It measured leisure participation and exploration based on the CMOP-E model, not only by doing leisure participation but also by having leisure time; the scope of leisure exploration was expanded so that measures were taken to improve the health and quality of life. Therefore, this study was significant in that it has established the foundation for academic and clinical development of leisure assessment for the elderly by applying multidimensional measurements on objective and subjective aspects of participation through the development of tools for evaluating elderly leisure based on practice models in occupational therapy.

The leisure participation assessment for the elderly showed high reliability of Cronbach's *α* value of 0.939 on all items, and the test-retest reliability was an assessment tool with high reliability with an interrater correlation coefficient of 0.941. However, Cronbach's alpha value of “Watch TV” of leisure participant among the results of internal consistency test for reliability was -0.43. Negative values mean a mixture of positive and negative responses. These results can be interpreted as having been the result of evenly responding responses to satisfaction of leisure participation, such as 2 or 3, to those who showed low satisfaction and those who showed high satisfaction by 9 or 10. According to a previous study, “Watching TV” was the most time-consuming activity of the elderly [6, 7, 37, 38]. The reason why the participation of the passive/static time-consuming leisure activities of the elderly in Korea was high was reported to differences in values of generations [39]. The modern generation of the Korean elderly was unable to properly experience leisure because they regarded it as an important life value to spend frugally and work diligently, and there was a lack of awareness and education about leisure because leisure was negatively perceived in the society [39]. For this reason, active leisure participation has not been achieved, and either time has been vaguely spent or a passive and simple leisure activity has become the mainstay [6, 31]. In previous study reports, these passive/static leisure activities were considered insignificant leisure activities comprising low-quality leisure activities [6, 38, 40]. However, in this study, a passive and time-consuming leisure activity such as “Watching TV” can be considered a meaningful activity if the elderly feels pleasure and satisfaction in “Watching TV” based on the results of internal consistency of leisure participation. In fact, according to data on the distribution of the response of “Watching TV,” the number of subjects who answered low satisfaction of 5 point or less was 9, and the number of subjects who answered 5 point was 17. And the number of subjects who answered high satisfaction of 5 point or more was 74. Thus, the assessment tool developed in this study to evaluate the elderly's leisure participation was meaningful in that, by measuring satisfaction with participating leisure activities, passive/static leisure activities can be relatively identified as a positive leisure time based on the elderly's emotional perception of the activity.

The Cronbach's *α* value for the reliability test of interference factors was 0.458, indicating low reliability. Previous studies showed that leisure participation is significantly associated with cultural capital, such as occupation, educational level, income level, and health status [36, 39]. The results of this study's interference factors are data that were responded by the elderly in some parts of the country, indicating the constraints of the individual's situation. Therefore, a further study of leisure restrictions based on urban and rural areas, gender, and age using a larger number of subjects is required.

In this study, we analyzed leisure participation and leisure exploration according to age to verify discriminant validity. The results showed that there were statistically significant differences in the number of participating leisure activities in the 60s, 70s, and 80s and participating leisure activities. These results were consistent with the results of the previous study showing that the desire for new leisure tends to decrease in old age, while static and passive leisure increases [32]. The reason for this is that modern-day Korean elderly and elderly people have very limited opportunity to experience leisure by living a labor-oriented society and past leisure consumption was closely associated with cultural capital such as job and educational level [39, 41]. Therefore, this assessment can be deduced that there was discriminating power in understanding the level of leisure participation and exploration according to the age of the elderly.

In 2017, Korea has entered an aged society with an aging rate of over 14%. The National Statistical Office estimates that Korea's aging population will continue to increase and enter the super-aged society with an aging rate of 20% in 2026 [42]. Population aging is a characteristic of the demographic structure experienced worldwide and can be explained by the increase in life expectancy due to economic growth and medical technology development [17]. It is important to maintain a meaningful age in accordance with the increased life expectancy, and it is necessary to have an occupational therapy approach to the leisure time, which occupies most of the elderly's time during the day. Occupational therapy in community-based rehabilitation plays an important role in integrating the elderly into the community. Previous studies have shown that community occupational therapies have an effect on the functional risk, functional ability, social participation, and quality of life of the elderly [43–45]. It is important to establish the occupational therapists' roles in the elderly's leisure activities and to evaluate the effectiveness of the elderly's leisure participation to develop the community occupational therapy services for the elderly. Therefore, the leisure participation assessment for the elderly developed in this study can contribute to the development of community-based occupational therapy.

The leisure participation assessment tool for the elderly developed in this study can be used by clinicians with the confirmation of the current level of leisure participation, leisure exploration, and interference factors of the elderly. It is possible to determine what leisure activities are currently involved in, how often they are done, and how satisfied they are with their participation through the evaluation of “Leisure participation.” “Leisure exploration” can provide the elderly with an opportunity to recognize interests, skills, opportunities, and appropriate leisure activities. “Interference factors” are factors that hinder leisure participation [46] suggested in the previous study: physical constraints, economic constraints, time constraints, information constraints, environmental constraints (transportation, facilities, etc.), and attitude constraints (constraints related to the individual's state of mind). It is possible to understand which factors affect the individual among, so that occupational therapists can consider the leisure participation and exploration of the elderly when planning interventions.

This study has some limitations. First, the generalization of this study was limited because this study only included the elderly residing in 3 provinces and excluded the elderly residing in other rural areas. Therefore, studies that assess the elderly's leisure participation and exploration in large-scale domestic elderly individuals are required. Second, the leisure participation assessment for the elderly developed in this study was not able to interpret the evaluation score because a scoring system for the leisure participation and leisure search of the elderly was not used. There were limitations in comparing the differences between prepost scores based on the total score. Therefore, it is necessary to establish a scoring system that can be interpreted using the total score so that the level of leisure participation can be clinically interpreted to evaluate the effectiveness of the interventions.

## 5. Conclusion

This study is aimed at developing standardized leisure participation assessment tool that could be useful in community-based occupational therapy and at developing efficient and useful tools that quantitatively and qualitatively measure leisure participation and exploration in the elderly. A total of 81 leisure items classified into 8 categories and 22 subcategories were obtained through data collection and Delphi survey. As a result of reliability and validity, highly validated and reliable leisure participation assessment for the elderly was confirmed. The leisure participation assessment for the elderly developed in this study can obtain information on the overall view of the leisure of the elderly by measuring leisure exploration, leisure participation, and interference factor affecting leisure participation. In addition, it will be an assessment that can obtain useful information by composing multidimensional measurements which can be both quantitative measurements such as frequency and qualitative measurements such as satisfaction and interest. Therefore, the leisure participation assessment tool for the elderly developed in this study could contribute to the expansion and development of the academic and clinical scope of community occupational therapy.

## Figures and Tables

**Table 1:** Comparison of the second and third Delphi survey results.

Delphi survey	Mean	SD	CVR	Convergence	Consensus	Stability
Second	4.02	0.86	0.48	0.55	0.72	0.22
Third	4.30	0.70	0.66	0.41	0.80	0.17

SD: standard deviation; CVR: content validity ratio.

**Table 2:** The final item of the leisure participation assessment for the elderly through the Delphi survey.

Item		Item	
1	Reading the Bible/Buddhist books	45	Collecting (stamps, coins, stones, etc.)
2	Reading a book	46	Shopping (for pleasure)
3	Reading a newspaper	47	Playing gate ball
4	Reading a magazine	48	Playing musical instruments (janggu, samulnori, saxophone, harmonica)
5	Viewing photos	49	Singing/singing folks
6	Creative writing	50	Yoga
7	Puzzles	51	Aerobics/dancing
8	Flower arranging	52	Racket exercise (tennis, badminton, table tennis, etc.)
9	Resting	53	Water exercises (swimming, aquarobic, etc.)
10	Painting	54	Learning foreign languages
11	Watching television	55	Korean chess/baduk
12	Listening to music	56	Dance sports
13	Listening to the radio	57	Hwatu/card games
14	Meditation	58	Drinking/dining with neighbors
15	Handicrafts (sewing, quilting, felt, embroidery, knitting, handicrafts, DIY, etc.)	59	Chatting with others
16	Mobile games	60	Attending book clubs
17	Computer/Internet (playing games, online shopping, searching)	61	Singing class
18	Using a phone (messenger, text, phone)	62	Volunteering
19	Spending time with pets	63	Visiting family/relatives/friends
20	Cooking (hobby)	64	Visiting cultural sites
21	Making healthy foods	65	Going to the cafe
22	Praying	66	Going to the bookstore/library
23	Calligraphy	67	Joining local festivals
24	Exercise bike	68	Eating out
25	Indoor exercises (stretching, strength exercise)	69	Going on a trip to enjoy flowers/maples
26	Gardening/growing flowers	70	Attending social clubs
27	Vegetable gardening	71	Attending health-related lectures (dementia, laughter treatment, etc.)
28	Walking	72	Computer/mobile class
29	Riding a bicycle	73	Mountain climbing
30	Visiting parks	74	Going to cinemas
31	Flower/tree viewing in town	75	Going to plays
32	Taking a walk with pets	76	Going to concerts
33	Visiting town hall	77	Watching musicals
34	Visiting the church/cathedral/temple	78	Going to exhibitions
35	Joining resident association activities	79	Watching sports games
36	Attending the village festival	80	Photography
37	Health gymnastics	81	Driving for pleasure
38	Using exercise equipment in the park	82	Domestic traveling (train, sightseeing bus)
39	Karaoke	83	Overseas trips
40	Participating in a senior university	84	Camping
41	Going to the sauna	85	Fishing
42	Going to the gym	86	Playing golf (outdoor)
43	Jogging	87	Boating (canoeing, sailing, cruise ships, etc.)
44	Screen golf		

**Table 3:** Items of leisure participation assessments for the elderly.

Category	Subcategories
Exercise	Exercise alone (walking, jogging, climbing, swimming, biking, aerobics, dumbbells, fitness, yoga, stretching, gymnastics, etc.)

Game	Exercise with more than two people or activities with skills and rules (golf, racket exercise, fishing, etc.)
	Board games (janggi, baduk, hwatu, card, etc.)
	Video games (computers, mobile games, etc.)

Social activity	Visiting (church, cathedral, temple, neighborhood, family, relatives, friends, etc.)
	Gathering (chatting with others, book clubs, eating out, visiting parks, sauna, going to a town hall, etc.)
	Participating in events (village festivals, local festivals, etc.)
	Volunteering
	Communicating (messenger, text, phone, email, etc.)

Culture	Art and creative activities (calligraphy, flower arranging, photography, creative writing, painting, music, handicraft, collecting, etc.)
	Gardening (vegetable gardening, gardening, growing flowers, etc.)
	Appreciation and watching (musicals, movies, exhibitions, sports games, plays, etc.)
	Reading (books, newspapers, magazines, Bibles, Buddhist books, etc.)

Learning	Attending in classes (senior university, foreign language, computer/mobile, health, etc.)

Refresh	Relaxation activities (resting, meditations, prayer, going to the café, etc.)

Outing	Traveling (domestic, overseas, going on a trip to enjoy flowers/maples, etc.)
	Camping
	Shopping (markets, department stores, marts)
	Driving

Information communication	Watching television
	Listening to the radio
	Using the Internet media (computer, mobile, etc.)

**Table 4:** The result of internal consistency (*N* = 100).

Items		Cronbach's *α*	Cronbach's *α* of items
Leisure participation	Exercise	.623	.911
	Sports	.736	
	Board games	.744	
	Video games	.736	
	Visiting	.719	
	Gathering	.553	
	Participating in events	.752	
	Volunteering	.673	
	Communicating	.790	
	Art and creative activity	.668	
	Gardening	.695	
	Appreciation and watching	.752	
	Reading	.771	
	Attending in classes	.709	
	Relaxation activity	.686	
	Traveling	.775	
	Camping	.753	
	Shopping	.733	
	Driving	.697	
	Watching television	-.043	
	Listening to the radio	.803	
	Using the Internet media	.800	

Leisure exploration	Exercise	.668	.921
	Sports	.728	
	Board games	.700	
	Video games	.765	
	Visiting	.762	
	Gathering	.692	
	Participating in events	.784	
	Volunteering	.653	
	Communicating	.728	
	Art and creative activity	.626	
	Gardening	.693	
	Appreciation and watching	.728	
	Reading	.713	
	Participating in classes	.657	
	Relaxation activity	.698	
	Traveling	.799	
	Camping	.846	
	Shopping	.791	
	Driving	.752	
	Watching television	.827	
	Listening to the radio	.942	
	Using the Internet media	.829	

Interference factor (8 items)			.458

Total Cronbach's *α* = .939			

*p* < 0.05.

**Table 5:** The result of content validity (*N* = 10).

Category	Subcategories	Item	I-CVI	S-CVI
Exercise	Exercise alone (walking, jogging, climbing, swimming, biking, aerobics, dumbbells, fitness, yoga, stretching, gymnastics, etc.)	1	1.00	0.95
Game	Exercise with more than two people or activities with skills and rules (golf, racket exercise, fishing, etc.)	2	0.80	
	Board games (janggi, baduk, hwatu, card, etc.)	3	1.00	
	Video games (computers, mobile games, etc.)	4	1.00	
Social activity	Visiting (church, cathedral, temple, neighborhood, family, relatives, friends, etc.)	5	1.00	
	Gathering (chatting with others, book clubs, eating out, visiting parks, sauna, going to a town hall, etc.)	6	1.00	
	Participating in events (village festivals, local festivals, etc.)	7	1.00	
	Volunteering	8	1.00	
	Communicating (messenger, text, phone, email, etc.)	9	1.00	
Culture	Art and creative activities (calligraphy, flower arranging, photography, creative writing, painting, music, handicraft, collecting, etc.)	10	1.00	
	Gardening (vegetable gardening, gardening, growing flowers, etc.)	11	0.80	
	Appreciation and watching (musicals, movies, exhibitions, sports games, plays, etc.)	12	1.00	
	Reading (books, newspapers, magazines, Bibles, Buddhist books, etc.)	13	1.00	
Learning	Attending in classes (senior university, foreign language, computer/mobile, health, etc.)	14	1.00	
Refresh	Relaxation activities (resting, meditations, prayer, going to the café, etc.)	15	0.80	
Outing	Traveling (domestic, overseas, going on a trip to enjoy flowers/maples, etc.)	16	1.00	
	Camping	17	1.00	
	Shopping (markets, department stores, marts)	18	0.90	
	Driving	19	0.90	
Information communication	Watching television	20	0.80	
	Listening to the radio	21	0.80	
	Using the Internet media (computer, mobile, etc.)	22	1.00	

**Table 6:** The result of discrimination validity (*N* = 100).

	Age	Mean ± SD	F	*P*	Post hoc (Scheffe)
The number of participating leisure activities	60s (a)	14.14 ± 3.58	71.182	.000	*c* < *b* < *a*
	70s (b)	7.51 ± 3.31			
	80s and above (c)	5.09 ± 1.73			
The number of activities of leisure exploration	60s (a)	5.60 ± 5.16	16.690	.000	*c*, *b* < *a*
	70s (b)	1.90 ± 2.42			
	80s and above (c)	1.12 ± 0.99			

*p* < 0.05.

## Data Availability

The data that support the findings of this study are available from the corresponding author upon reasonable request.

## Supplementary Materials

Supplementary Materials Supplementary Table S1: leisure participation. “Leisure” refers to voluntary activities involving free time in addition to compulsory activities such as work, self-care, housework, or sleep. Please write down the leisure activities you are participating in, and indicate the frequency of participation and satisfaction. Table S2: leisure exploration. If you have any leisure activities you would like to participate in other than your current leisure activities, please write down the leisure activities, and indicate your intention (how often you want to participate) and interest. Table S3: interference factors. Indicate the factors that limit your leisure participation.
