# Supplementary material for: The Development of Leisure Participation Assessment Tool for the Elderly
Source: Occup Ther Int. 2020 Dec 4;2020:9395629. doi: 10.1155/2020/9395629 (PMC7787791; doi:10.1155/2020/9395629)
Supplement: Supplementary Materials — Supplementary Table S1: leisure participation. “Leisure” refers to voluntary activities involving free time in addition to compulsory activities such as work, self-care, housework, or sleep. Please write down the leisure activities you are participating in, and indicate the frequency of participation and satisfaction. Table S2: leisure exploration. If you have any leisure activities you would like to participate in other than your current leisure activities, please write down the leisure activities, and indicate your intention (how often you want to participate) and interest. Table S3: interference factors. Indicate the factors that limit your leisure participation. [file 9395629.f1.docx]

**Table S1. Leisure participation**

“Leisure” refers to voluntary activities involving free time in addition to compulsory activities such as work, self-care, housework or sleep. Please write down the leisure activities you are participating in, and indicate the frequency of participation and satisfaction.

| Category | Subcategories | Activity | Participation | Satisfaction | |
| --- | --- | --- | --- | --- | --- |
| Exercise | Exercise alone (walking, jogging, climbing, swimming, biking, aerobics, dumbbells, fitness, yoga, stretching, gymnastics, etc.) |  | No□ Weekly□ Monthly□ Annually□ Frequency ____ | | _____/10 |
| Game | Exercise with more than two people or activities with skills and rules (golf, racket exercise, fishing, etc.) |  | No□ Weekly□ Monthly□ Annually□ Frequency ____ | _____/10 | |
|  | Board games (janggi, baduk, hwatu, card, etc.) |  | No□ Weekly□ Monthly□ Annually□ Frequency ____ | _____/10 | |
|  | Video games (computers, mobile games, etc.) |  | No□ Weekly□ Monthly□ Annually□ Frequency ____ | _____/10 | |
| Social activity | Visiting (church, cathedral, temple, neighborhood, family, relatives, friends, etc.) |  | No□ Weekly□ Monthly□ Annually□ Frequency ____ | _____/10 | |
|  | Gathering (chatting with others, book clubs, eating out, visiting parks, sauna, going to a town hall, etc.) |  | No□ Weekly□ Monthly□ Annually□ Frequency ____ | _____/10 | |
|  | Participating in events (village festivals, local festivals, etc.) |  | No□ Weekly□ Monthly□ Annually□ Frequency ____ | _____/10 | |
|  | Volunteering |  | No□ Weekly□ Monthly□ Annually□ Frequency ____ | _____/10 | |
|  | Communicating (messenger, text, phone, email, etc.) |  | No□ Weekly□ Monthly□ Annually□ Frequency ____ | _____/10 | |
| Culture | Art and creative activities (calligraphy, flower arranging, photography, creative writing, painting, music, handicraft, collecting, etc.) |  | No□ Weekly□ Monthly□ Annually□ Frequency ____ | _____/10 | |
|  | Gardening (vegetable gardening, gardening, growing flowers, etc.) |  | No□ Weekly□ Monthly□ Annually□ Frequency ____ | _____/10 | |
|  | Appreciation and watching (musicals, movies, exhibitions, sports games, plays, etc.) |  | No□ Weekly□ Monthly□ Annually□ Frequency ____ | _____/10 | |
|  | Reading (books, newspapers, magazines, Bibles, Buddhist books, etc.) |  | No□ Weekly□ Monthly□ Annually□ Frequency ____ | _____/10 | |
| Learning | Attending in classes (senior university, foreign language, computer/mobile, health, etc.) |  | No□ Weekly□ Monthly□ Annually□ Frequency ____ | _____/10 | |
| Refresh | Relaxation activities (resting, meditations, prayer, going to the café, etc.) |  | No□ Weekly□ Monthly□ Annually□ Frequency ____ | _____/10 | |
| Outing | Traveling (domestic, overseas, Going on a trip to enjoy flowers/maples, etc.) |  | No□ Weekly□ Monthly□ Annually□ Frequency ____ | _____/10 | |
|  | Camping |  | No□ Weekly□ Monthly□ Annually□ Frequency ____ | _____/10 | |
|  | Shopping (markets, department stores, marts) |  | No□ Weekly□ Monthly□ Annually□ Frequency ____ | _____/10 | |
|  | Driving |  | No□ Weekly□ Monthly□ Annually□ Frequency ____ | _____/10 | |
| Information communication | Watching television |  | No□ Weekly□ Monthly□ Annually□ Frequency ____ | _____/10 | |
|  | Listening to the radio |  | No□ Weekly□ Monthly□ Annually□ Frequency ____ | _____/10 | |
|  | Using the Internet media (computer, mobile, etc.) |  | No□ Weekly□ Monthly□ Annually□ Frequency ____ | _____/10 | |

**Table S2. Leisure exploration**

If you have any leisure activities you would like to participate in other than your current leisure activities, Please write down the leisure activities, and indicate your intention (How often you want to participate) and interest.

| Category | Subcategories | Activity | Participation intention | Interest | |
| --- | --- | --- | --- | --- | --- |
| Exercise | Exercise alone (walking, jogging, climbing, swimming, biking, aerobics, dumbbells, fitness, yoga, stretching, gymnastics, etc.) |  | No□ Weekly□ Monthly□ Annually□ Frequency ____ | | _____/10 |
| Game | Exercise with more than two people or activities with skills and rules (golf, racket exercise, fishing, etc.) |  | No□ Weekly□ Monthly□ Annually□ Frequency ____ | _____/10 | |
|  | Board games (janggi, baduk, hwatu, card, etc.) |  | No□ Weekly□ Monthly□ Annually□ Frequency ____ | _____/10 | |
|  | Video games (computers, mobile games, etc.) |  | No□ Weekly□ Monthly□ Annually□ Frequency ____ | _____/10 | |
| Social activity | Visiting (church, cathedral, temple, neighborhood, family, relatives, friends, etc.) |  | No□ Weekly□ Monthly□ Annually□ Frequency ____ | _____/10 | |
|  | Gathering (chatting with others, book clubs, eating out, visiting parks, sauna, going to a town hall, etc.) |  | No□ Weekly□ Monthly□ Annually□ Frequency ____ | _____/10 | |
|  | Participating in events (village festivals, local festivals, etc.) |  | No□ Weekly□ Monthly□ Annually□ Frequency ____ | _____/10 | |
|  | Volunteering |  | No□ Weekly□ Monthly□ Annually□ Frequency ____ | _____/10 | |
|  | Communicating (messenger, text, phone, email, etc.) |  | No□ Weekly□ Monthly□ Annually□ Frequency ____ | _____/10 | |
| Culture | Art and creative activities (calligraphy, flower arranging, photography, creative writing, painting, music, handicraft, collecting, etc.) |  | No□ Weekly□ Monthly□ Annually□ Frequency ____ | _____/10 | |
|  | Gardening (vegetable gardening, gardening, growing flowers, etc.) |  | No□ Weekly□ Monthly□ Annually□ Frequency ____ | _____/10 | |
|  | Appreciation and watching (musicals, movies, exhibitions, sports games, plays, etc.) |  | No□ Weekly□ Monthly□ Annually□ Frequency ____ | _____/10 | |
|  | Reading (books, newspapers, magazines, Bibles, Buddhist books, etc.) |  | No□ Weekly□ Monthly□ Annually□ Frequency ____ | _____/10 | |
| Learning | Attending in classes (senior university, foreign language, computer/mobile, health, etc.) |  | No□ Weekly□ Monthly□ Annually□ Frequency ____ | _____/10 | |
| Refresh | Relaxation activities (resting, meditations, prayer, going to the café, etc.) |  | No□ Weekly□ Monthly□ Annually□ Frequency ____ | _____/10 | |
| Outing | Traveling (domestic, overseas, Going on a trip to enjoy flowers/maples, etc.) |  | No□ Weekly□ Monthly□ Annually□ Frequency ____ | _____/10 | |
|  | Camping |  | No□ Weekly□ Monthly□ Annually□ Frequency ____ | _____/10 | |
|  | Shopping (markets, department stores, marts) |  | No□ Weekly□ Monthly□ Annually□ Frequency ____ | _____/10 | |
|  | Driving |  | No□ Weekly□ Monthly□ Annually□ Frequency ____ | _____/10 | |
| Information communication | Watching television |  | No□ Weekly□ Monthly□ Annually□ Frequency ____ | _____/10 | |
|  | Listening to the radio |  | No□ Weekly□ Monthly□ Annually□ Frequency ____ | _____/10 | |
|  | Using the Internet media (computer, mobile, etc.) |  | No□ Weekly□ Monthly□ Annually□ Frequency ____ | _____/10 | |

**Table S3. Interference factors**

Indicate the factors that limit your leisure participation.

|  | Interference factors | very likely | somewhat likely | neutral | somewhat unlikely | very unlikely |
| --- | --- | --- | --- | --- | --- | --- |
| 1 | Physical problems | ① | ② | ③ | ④ | ⑤ |
| 2 | Cost problems | ① | ② | ③ | ④ | ⑤ |
| 3 | Lack of time | ① | ② | ③ | ④ | ⑤ |
| 4 | Lack of information related to leisure | ① | ② | ③ | ④ | ⑤ |
| 5 | Inconvenience of using transportation | ① | ② | ③ | ④ | ⑤ |
| 6 | Lack of leisure facilities | ① | ② | ③ | ④ | ⑤ |
| 7 | No need for leisure | ① | ② | ③ | ④ | ⑤ |
| 8 | Other: | ① | ② | ③ | ④ | ⑤ |
